# Bibliography

**Published:** 1889-02

**Authors:** 


### BIBLIOGRAPHY.

We desire to acknowledge the receipt of two reprints from Dr. George Cunningham: *A Professional Holiday*, being an account of his visit to America last year and his impressions of American dentists as he saw them in the Ninth Medical Congress and his paper on the *Treatment of Pulpless and Abscessed Teeth.* The Dr. is an able and concise writer, and in his "Holiday" notes has also shown himself a versatile writer.

An elaborate work upon the status of dentistry in America by Dr. Kuhn is also at hand. It is in the form of a report to the Board of Public Instruction in Paris, and treats of our colleges, our State laws, and our societies. In a supplement the author also considers the institutions of England, her colonies and the Continent. The book is fully illustrated with cuts of our colleges and is, so far as we are aware, the first and only effort that has been made to compile statistics in regard to our dental institutions. The Board should feel thankful that they secured the services of so able and indefatigable a worker as Dr. Kuhn.

Clinical Lecture on Certain Diseases of the Nervous System. By J. M. Charcot, M.D. Translated by E. P. Hurd, M.D.

Prof. Charcot is perhaps one of the most voluminous writers in the medical profession, having published up to the present time upwards of two hundred brochures, memoirs, and books. As a thinker and original writer he is well known. His investigations have always been far-reaching, and the subjects of interest. As an histologist and pathologist he has ranked high for the past fifty years. His work on the spinal cord, in which he differentiated the nervous from the connective tissue elements, was unrivaled at the time. His subsequent labors on sclerosis alone are sufficient to perpetuate his name as an original investigator. The present volume should be in the hands of every dentist, treating as it does of psychological as well as nervous disorders, both of which come under our notice in practice, and about which we should know more.

We desire to call attention to another book of similar character by J. Leonard Corning, which treats of hysteria and epilepsy. The subject of hysteria in women and children is particularly well handled, and is a subject about which dentists can not know too much.

These books belong to the Physicians' Leisure Library Series, a third of which is also at hand, treating of abdominal surgery, by H. C. Wyman, M.D. The series is published by George S. Davis, Detroit, Michigan. $2.50 per annum, issued monthly; 25 cents single copies. Cloth, 50 cents.

Photographic Illustrations of Skin Diseases. By George Henry Fox, A.M., M.D. New York. E. B. Treat, Publisher, 771 Broadway.

Parts seven and eight of this magnificent work have been received, and are quite equal in interest to any that have preceded them. We cannot well say more in their favor to any one at all interested in dermatology. The work is of the utmost importance, for each one of the numerous plates gives a clinical lesson which cannot be mistaken.

We have received from Dr. H. W. Harkness a neat sixty-four page pamphlet, descriptive of the Leland Stanford Junior University of California. The book is illustrated with photogravure plates of the board of trustees and those intimately connected with the work of founding the institution. This is a novel method of presenting the university before the public, and one that could be adopted to advantage by some of our eastern institutions. If we were more familiar with the faces of the benefactors and advisors of our educational institutions, we would feel a greater interest in their advancement.

